# Effects of Cognitive Behavioral Therapy on Pain and Sleep in Adults with Traumatic Brain Injury: A Systematic Review and Meta-Analysis

**DOI:** 10.1155/2021/6552246

**Published:** 2021-11-11

**Authors:** Xin Li, Yuwei Feng, Jianping Xia, Xuan Zhou, Nan Chen, Zhengquan Chen, Qimeng Fan, Hong Wang, Peiyuan Ding, Qing Du

**Affiliations:** ^1^Department of Rehabilitation, Xinhua Hospital, School of Medicine, Shanghai Jiaotong University, Shanghai 200092, China; ^2^School of Kinesiology, Shanghai University of Sport, Shanghai 200438, China; ^3^Department of Rehabilitation, Maternity & Child Care Center of Xinyu, Xinyu, 338000 Jiangxi, China; ^4^Department of Rehabilitation, Chongming Branch of Xinhua Hospital, School of Medicine, Shanghai Jiaotong University, Shanghai 202150, China; ^5^College of Rehabilitation Science, Shanghai University of Medicine & Health Sciences, Shanghai 201318, China; ^6^Department of Neurosurgery, Chongming Branch of Xinhua Hospital, School of Medicine, Shanghai Jiaotong University, Shanghai 202150, China

## Abstract

The objective of this study was to systematically review the literature on the effects of cognitive behavioral therapy (CBT) on insomnia and pain in patients with traumatic brain injury (TBI). PubMed, Embase, the Cochrane Library, Cumulative Index to Nursing and Allied Health, and Web of Science databases were searched. Outcomes, including pain, sleep quality, and adverse events, were investigated. Differences were expressed using mean differences (MDs) with 95% confidence intervals (CIs). The statistical analysis was performed using STATA 16.0. Twelve trials with 476 TBI patients were included. The included studies did not indicate a positive effect of CBT on pain. Significant improvements were shown for self-reported sleep quality, reported with the Pittsburgh Self-Reported Sleep Quality Index (MD, -2.30; 95% CI, -3.45 to -1.15; *P* < 0.001) and Insomnia Severity Index (MD, -5.12; 95% CI, -9.69 to -0.55; *P* = 0.028). No major adverse events related to CBT were reported. The underpowered evidence suggested that CBT is effective in the management of sleep quality and pain in TBI adults. Future studies with larger samples are recommended to determine significance. This trial is registered with PROSPERO registration number CRD42019147266.

## 1. Introduction

Traumatic brain injury (TBI) is a global public health and medical priority with an annual incidence estimated at 200~1967 per 100,000 of the population [1]. The age-standardized prevalence of TBI increased by 8.4% from 1990 to 2016 globally [2, 3], and it became the third leading cause of death and disability [4]. Regardless of the extent, both acute and more chronic consequences that lead to permanent behavioral disabilities and pain associated with most TBIs are due to diffuse axonal injury [5]. Approximately 65% of patients who survive moderate-to-severe TBI subsequently suffer from a wide range of symptoms ranging from physical disabilities (pain, fatigue, etc.) to psychological impairments (hypomnesia, depression, anxiety, etc.) [6], which reduces life expectancy and presents a substantial economic burden to victims, their families, and society as a whole [7].

Pain is reported in over 50% of TBI patients [8], with approximately 20% of TBI patients developing possible neuropathic pain and sleep problems [9]. In most cases, pain is associated with other post-TBI complaints [10], such as sleep disturbance, which represents a vital interventional target, although sleep disorders and pain are sometimes two independent and separately occurring symptoms of TBI. To enhance interventional efficacy, particularly for TBI patients who have severe pain [11], cognitive behavioral therapies should target both sleep and pain due to the negative effect of pain on sleep quality.

Strong evidence has shown that cognitive behavioral therapy (CBT) is beneficial for the nonbrain-injured population that has cognitive impairments, such as those with anxiety, depression, or intellectual disabilities [12, 13], or for the population with acquired brain injuries, such as those who experienced cerebral vascular accident, anoxia, and neurosurgery [14]. The mechanisms underpinning these improvements appear to be that CBT helps TBI patients understand how to identify and change disturbing thought patterns that have a negative influence on behavior and emotions through a psychotherapeutic approach [15, 16]. Therefore, CBT is an alternative option for patients who suffer from pain and are not suitable for drug therapy. The evidence suggests that CBT, as one of the neuropsychological interventions that combines cognitive and behavioral techniques [17], is the “gold standard” treatment for pain-related symptoms in those with a wide range of musculoskeletal or neurological diseases [18]. However, there has been conjecture that CBT is also effective in post-TBI pain (headache or widespread pain). Moreover, CBT has also been recommended as a first-line treatment for other pain-related dysfunctions (such as sleep disorders and neuropathic pain) [19], although no quantitative meta-analysis has been performed to investigate the effects of CBT on sleep quality in adults with TBI. The present systematic review with meta-analysis is therefore aimed at examining the evidence for the effectiveness of CBT programs on pain and sleep quality in patients with TBI.

## 2. Methods

### 2.1. Literature Search and Selection Criteria

This meta-analysis was planned, conducted, and reported in adherence with the Preferred Reporting Items for Systematic Review and Meta-Analysis (PRISMA) statement [20]. Using search terms such as “traumatic brain injury”, “TBI”, “cognitive behavio(u)r therapy∗”, “CBT”, “pain” and “sleep”, we searched PubMed, Embase, the Cochrane Library, Cumulative Index of Nursing and Allied Health Literature (CINAHL), and Web of Science for English-language parallel-group studies reporting the effect of CBT in TBI patients published up to July 2021. The search strategies are shown in Appendix S1. Two reviewers (Peiyuan D and Qing D) independently performed the systematic literature search, detected and deleted all duplicate records, screened the titles, and identified abstracts based on relevance. The full-text articles designated for inclusion were reviewed. In addition, the reference lists of the retrieved articles and available review articles were manually checked to identify additional eligible studies.

Studies were selected for detailed review if they fulfilled the following population, intervention, comparison, outcome, and study design (PICOs) framework: (1) population: TBI participants who had brain damage due to external forces (such as direct impact, rapid acceleration or deceleration, a penetrating injury, or blast waves from an explosion) or a subgroup with TBI whose data could be extracted by the authors, with no restrictions on age, sex, or ethnicity (regular medication use was allowed); (2) intervention: any treatment classified as CBT; (3) comparison: no treatment or non-CBT (including pharmacotherapy); and (4) outcomes: primary outcomes were pain (measured by visual analog scales, the McGill Pain Questionnaire (MPQ), a pain diary, or pressure pain thresholds), sleep quality (assessed by the self-reported Pittsburgh Sleep Quality Index (PSQI), and adverse events associated with CBT, which were reported as the number of participants experiencing any adverse event, number of participants who withdrew because of adverse events, and number of participants experiencing any serious adverse event. Data from randomized controlled trials (RCTs) and case studies were extracted, while only data from RCTs were synthesized.

### 2.2. Data Extraction and Quality Assessment

Data were extracted by Xin L using a customized data extraction form and independently confirmed by another reviewer (Yuwei F). Detailed information was extracted from each study, including first author, year of publication, study design, number of participants (% women), and demographic and outcome data. Detailed descriptions of the CBT intervention and control group in these RCTs were collected. When the same patients were reported in several publications, we retained only the publication with the largest sample size to avoid duplication of information. Discrepancies were resolved through discussion with a third reviewer (Jianping X) to reach a consensus. The Cochrane risk of bias tool [21] was used to assess the methodological quality of the included studies.

### 2.3. Statistical Analysis and Data Synthesis

Statistical analysis was performed using STATA, version 16.0. In the quantitative data synthesis section, a random effects model was chosen if two or more trials evaluated the same outcome in comparable groups with the mean difference (MD) and 95% confidence interval (CI) calculated for the summary statistics. If two or more control groups received various treatments in one trial, we combined the data from the control groups using the formula recommended by the Cochrane Handbook for Systematic Reviews of Interventions [21]. The median, interquartile range, and sample size of each trial were obtained to estimate the mean and variance for each study using simple and elementary inequalities and approximations if necessary [22]. The *I*^2^ statistic was calculated to assess heterogeneity among studies, with values < 25% indicating no heterogeneity, 25% to 50% indicating low heterogeneity, 50% to 75% indicating moderate heterogeneity, and >75% indicating high heterogeneity.

The potential publication bias was visually assessed by drawing a funnel plot. Additionally, corresponding authors were contacted to provide details on unreported data, which was required for our meta-analysis. The Grading of Recommendations Assessment, Development, and Evaluation (GRADE) system was applied to specify the quality of each outcome by categorizing studies into four levels (high, moderate, low, and very low) by accessing the following factors: study design, study limitations (risk of bias), inconsistency, indirectness of study results, imprecision, and publication bias [23] (shown in Appendix S2).

## 3. Results

### 3.1. Study Identification and Selection

**Figure 1:**
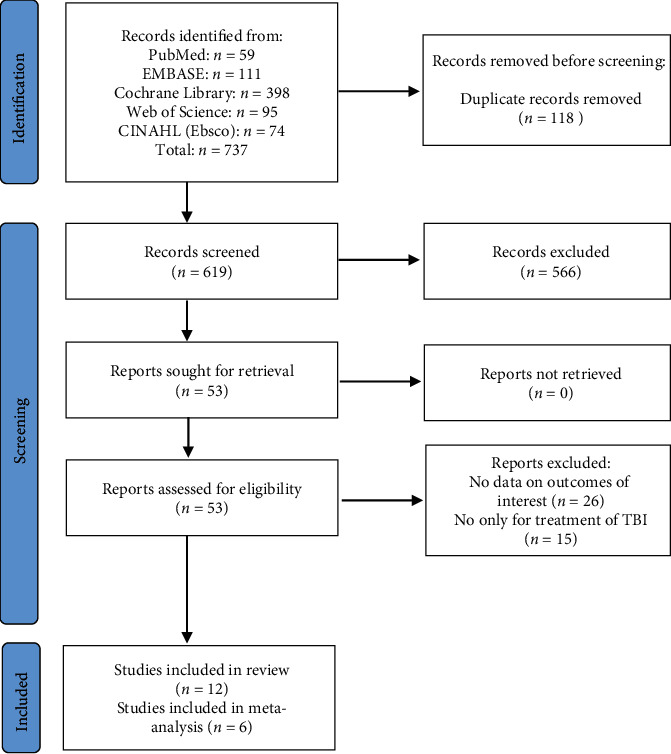
PRISMA flow diagram of studies in this review.

The initial electronic search returned a total of 737 records, with 619 unique records identified after duplicates were excluded. A total of 566 titles and abstracts were excluded for various reasons (i.e., they were reviews, letters, or irrelevant to the analysis). Of the remaining 53 articles, 6 RCTs and 6 case studies covering 476 patients were included based on the inclusion criteria.  Figure 1 shows the PRISMA flow diagram of the studies in this review.

### 3.2. Study Characteristics

The demographic and baseline clinical variables of the included studies are shown in Table 1. Studies included both sexes, the mean age of the subjects in the study ranged from 11 to 72 years, and the number of participants in the CBT group ranged from 1 to 200. All studies analyzed in this review included individuals with TBI.  Table 2 summarizes the detailed CBT methods in the intervention groups in the RCTs and case studies and the interventional methods in the control groups in the RCTs. Overall, the study duration lasted from 4 weeks to 1 year, with a median of 8 weeks and 4 to 12 sessions. The standard CBT protocol was mentioned in 3 RCTs [24–26] and 1 case study [27], while cognitive behavioral therapy for insomnia (CBT-I) was used in 1 RCT [28] and 4 case studies [29–32]. Two RCTs [33, 34] and 1 case series [35] implemented a CBT-based integrated intervention. An education intervention [26], a wait-list control condition [24, 25], or treatment as usual [28, 33, 34] was conducted in the control groups.

### 3.3. Quality Assessment

The assessment of the risk of bias for the included RCTs is shown in Table 3. All RCTs reported the numbers and reasons for withdrawal or dropout. Five of the included RCTs generated an adequately randomized sequence [24, 26, 28, 33, 34], and three were conducted in a blinded fashion for the outcome assessment [28, 33, 34]. Given that the pooled number of trials in this comparison was quite small (maximum of 3 trials), no funnel plot analysis was performed.

### 3.4. Outcome Measurements

#### 3.4.1. Primary Outcomes

*(1) Pain*. While pain is the main symptom after TBI and has a great impact on quality of life, only 4 RCTs and 3 case studies screened the severity of pain in various forms [24, 25, 33, 34]. Nguyen et al. [34] mentioned that the Brief Pain Inventory data of their participants were obtained at baseline; however, the Brief Pain Inventory was not assessed after the intervention. One of the RCTs used the MPQ [25] to quantify the severity of pain before and after the intervention and found no significant changes after the CBT intervention. Pressure pain thresholds and data from a headache diary were employed as outcome measures in Kjeldgaard and colleague's study, and there was no significant reduction in pain [24]. Moreover, the other RCT [33] used headache pain items from the Traumatic Brain Injury-Quality of Life questionnaire, and no significant improvement in pain was found. Because the three included RCTs [24, 25, 33] had a high degree of heterogeneity in the pain measurements, a meta-analysis of data may have been unconvincing.

In two of the case studies [27, 35], qualitative measures, such as the intensity and frequency of headache and medication use, were recorded at baseline and after a long-term follow-up (from 36 weeks to over one year), and significant improvements were found in the characteristics of the headaches, and much fewer pain killers were used. The Brief Pain Inventory was also used in the study of Lu et al.; however, the effects of CBT on the Brief Pain Inventory scores were contradictory [31].

**Figure 2:**
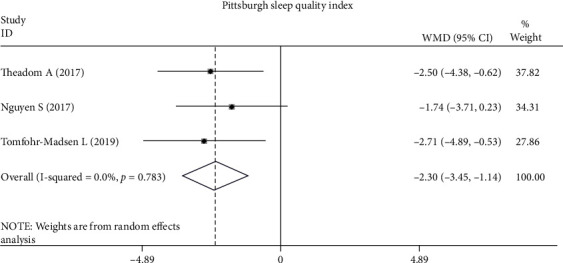
Differences in Pittsburgh Self-Reported Sleep Quality Index scores following CBT compared with other forms of interventions.

**Figure 3:**
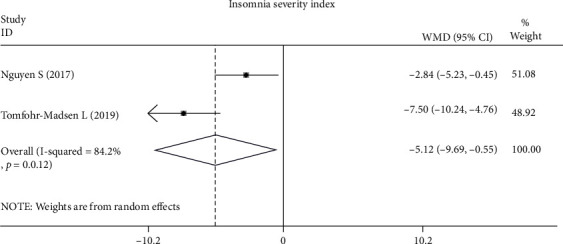
Differences in Insomnia Severity Index scores following CBT compared with other forms of interventions.

*(2) Sleep Quality*. Sleep quality was assessed in 4 RCTs [26, 28, 33, 34] and 4 case studies [29–32]. The PSQI is a self-reported questionnaire that demonstrates good psychometric properties for measuring sleep quality and impairment in various populations [26, 28, 34]. The pooled analysis across three studies [26, 28, 34] showed a significant improvement in self-reported sleep quality in the CBT group (MD, -2.30; 95% CI, -3.45 to -1.15; *P* < 0.001). The heterogeneity among studies was acceptable (*χ*^2^ = 0.49, *P* = 0.783, *I*^2^ = 0%) (Figure 2). The Insomnia Severity Index was used in Nguyen et al.'s [34] and Tomfohr-Madsen et al.'s study [28], and the pooled analysis showed that insomnia was significantly improved in the CBT group (MD, -5.12; 95% CI, -9.69 to -0.55; *P* = 0.028), but the heterogeneity among studies was high (*χ*^2^ = 6.31, *P* = 0.012, *I*^2^ = 84.2%) (Figure 3). Actigraphy, a validated objective test of sleep quality [36] used in Theadom et al.'s study [26], evaluates sleep onset, time awake, and the number of awakenings. However, there were no significant differences in the actigraphy measures after 6 weeks of a CBT-based online intervention. Additionally, in the two RCTs that recruited adolescents [28, 33], positive changes were also found in other sleep quality measures, such as the Dysfunctional Beliefs and Attitudes about Sleep Scale, a sleep diary, and the Adolescent Sleep Wake Scale.

The Insomnia Severity Index was used in all 4 case studies [29–32], and most of the participants showed a negative trend in the Insomnia Severity Index scores, which indicated improvements in insomnia, although the decrease in insomnia severity was not clinically significant in the study of Lu et al. [31]. Sleep diaries were another useful tool for recording daily sleep habits, and quantified data from sleep diaries, such as total sleep time and sleep efficiency, showed positive changes in the TBI participants with sleep disturbances in the 4 case studies [29–32].

*(3) Adverse Events*. CBT was well tolerated among the participants in most included studies. An average of 5.3 participants withdrew during the CBT intervention, and the overall dropout rate was 7.8% in the 6 included RCTs, mainly due to loss to follow-up or active withdrawal. Furthermore, no major adverse events, such as progression of symptoms, suicide, or death, were reported among the participants during the CBT intervention.

## 4. Discussion

The primary purpose of this meta-analysis was to examine the relative efficacy between CBT treatments and non-CBT treatments for TBI. The principal finding of this systematic review and meta-analysis of TBI was that CBT is associated with a significant improvement in self-reported sleep quality but not pain, and CBT was found to be well tolerated among these patients.

Due to the heterogeneity in pain evaluation methods across studies, a meta-analysis could not be performed, although the general trend of the results on pain was described. The TBI patients in the CBT groups did not have significant changes in pain or headache measured by questionnaires or a hand-held pressure algometer after the entire intervention in the included RCTs [24, 25, 33]. Contrary to the expectations that CBT would have marked efficacy on pain, even slight changes could not be discriminated considering the placebo effect of CBT. The reason for the lack of significance may be that most of the included patients suffered from long-term TBI sequelae, and the pain experience in the TBI patients may be profound and chronic. A neuroimaging study showed that chronic pain would remodel sensorimotor activation in the gray matter of the brain, such as widespread alterations in somatosensory cortices, supplementary motor areas, and superior temporal gyri [37]. It is estimated that if CBT or pain education is employed in the early stage after brain trauma, pain symptoms may not enter the chronic stage [24], while pharmacological therapy seems more effective in chronic pain [38]. In contrast to the results from the RCTs, a decrease in the intensity and frequency of headache was found in the two case studies [27, 35]. The mechanisms of the effects of CBT on pain relief lie in changing thoughts as they relate to pain and improving pain through behavioral reinforcement. These improvements require long-term CBT treatment. The CBT interventions in the case studies lasted for a long time, and the main intervention target was headache, so there was a positive intervention effect. Sleep disorders, which might be associated with diffuse axonal injury resulting in damage to sleep-regulating structures and disruptions in hypocretin-1, can be categorized: insomnia was found in 29%, hypersomnia in 28%, and sleep apnea in 25% of patients who have a history of TBI [39]. Sleep has a significant impact on the quality of life of TBI patients, and sleep disturbances have been consistently related to anxiety, depression, fatigue, or other complications. Many studies have reported that PSQI scores are associated with subjective questionnaire scores for anxiety and depression [40–42]. Although evidence on CBT specific to patients with TBI was scarce, our meta-analysis found a significant improvement in self-reported sleep quality measured with the PSQI, which was in accordance with the results of Ouellet MC's study [43]. However, there were no significant changes in actigraphy measures. Sleep quality is more like a subjective experience, and CBT could subjectively change participants' thoughts in relation to sleep and improve sleep behavior. As a result, self-reported sleep quality rose, and the objective data (actigraphy measures) may not improve as much as the subjective measures. Greater heterogeneity appeared in the data synthesis of the Insomnia Severity Index. In the two included RCTs, there were great difference characteristics of the participants, as female adolescents accounted for 75% of the participants in Tomfohr-Madsen et al.'s study [28], while the age span of the participants in Nguyen et al.'s study [34] was large. However, the biggest contributor to the heterogeneity was from the difference in baseline symptoms of insomnia. Insomnia in the participants in Tomfohr Madsen et al.'s study [28] was more severe than that in Nguyen et al.'s study [34], and CBT is known to achieve larger effect sizes in groups with more severe insomnia. To a certain extent, our results were partially contrary to Ford et al.'s conclusion that there was a reliable effect in support of CBT for TBI patients with sleep disorders [44]. Several methodological differences may be proposed to explain the contrasting findings. Whereas Ford and colleagues included 7 trials, comprising both clinical trials and single case studies, the present meta-analysis included only RCTs and was more concentrated on CBT and TBI. Last but not least, this was the first meta-analysis that synthesized evidence using quantitative methods, which provided a more objective estimate of the treatment effects.

## 5. Strengths and Limitations

Although CBT is recommended for treating pain and sleep disorders in people after TBI, there has been no systematic review that revealed the therapeutic effects of CBT. This systematic review and meta-analysis is the first to show the therapeutic effect of CBT on posttraumatic pain, especially headache. Sleep quality and insomnia symptoms were also significantly improved. However, there are several limitations in this study. First, we had only a limited number of clinical trials that assessed the efficacy and safety of CBT among patients with TBI; thus, publication bias cannot be completely ruled out. Second, only half of the included studies evaluated quality of life, and none of the included studies assessed TBI-related restrictions to participation in daily life. Third, as the included studies reported outcomes by various methods, it was relatively difficult to derive a powerful synthesis of data evaluating CBT in groups of patients with TBI. Finally, although the meta-analysis showed that there were significant changes in sleep quality and insomnia, the clinical importance of the changes may be limited. Future multicenter, well-designed, large, population-based randomized control trials are needed.

## 6. Conclusions

CBT is relatively safe and is associated with significant improvements in self-reported sleep quality among patients with TBI, while limited evidence has shown that pain cannot be significantly improved by CBT. Nevertheless, interpretation of our results must be done cautiously considering the methodological drawbacks and poor quality of the data in the included trials. Future studies with more homogeneous, objective assessments are needed to determine whether CBT can be used to improve long-term clinical endpoints among these patients in the real world.

## Figures and Tables

**Table 1:** Baseline demographic and clinical characteristics of study participants.

	Author (year)	Study type	No. of participants (% women)	Age (y): range/mean (SD)	Time since injury mean (SD)	Severity	Outcome measures	Adverse events	Time points	Dropout rate after intervention
1	McCarty et al., 2021 [33]	Randomized controlled trial	*T*: 200 (62.0) *I*: 101 (59.4) C:99 (69.6)	*T*: 14.7 (1.7) 11 to 18 *I*: 14.8 (1.7) *C*: 14.7 (1.7)	*I*: 0-30 days: 2 31-60 days: 62 61-90 days: 19 91-120 days: 8 121-180 days: 5 181-270 days: 5 *C*: 0-30 days: 4 31-60 days: 55 61-90 days: 17 91-120 days: 13 121-180 days: 5 181-270 days: 5	*T*: Mild:200	(1) Postconcussive symptoms (the Health Behavior Inventory) (2) Health-related quality of life (the Pediatric Quality of Life Inventory) (3) Psychological assessment: (the Patient Health Questionnaire–9, Generalized Anxiety Disorder-7 item scale, the 15-item anxiety subscale of the Revised Child Anxiety and Depression Scale-Short version) (4) Sleep quality (Adolescent Sleep Wake Scale) (5) Headache pain (Traumatic Brain Injury-Quality of Life headache pain)	None	*I*: baseline; 6-month intervention; 6-month follow-ups *C*: baseline; 6 months; 6-month follow-ups	*I*: 1.98% *C*: 4.04% *T*: 3.00%
2	Tomfohr-Madsen et al., 2019 [28]	Randomized controlled trial	*T*: 24 (75.0) *I*: 12 (75.0) *C*: 12 (75.0)	*T*: 15.0 (1.4) 12 to 18 *I*: 15.2 (1.5) *C*: 14.9 (1.3)	*T*: 1 month to 12 months	*T*: Mild: 24	(1) Sleep disturbance (Insomnia Severity Index, Pittsburgh Sleep Quality Index, Dysfunctional Beliefs and Attitudes about Sleep Scale, sleep diary) (2) Postconcussive symptoms (the Health Behavior Inventory) (3) Psychological assessment (Patient-Reported Outcomes Measurement Information System (PROMIS) Anxiety and Depression)	None	*I*: baseline; 6-week intervention; 4-week follow-ups *C*: baseline; 6 weeks; 4-week follow-ups	*I*: 16.67% *C*: 8.33% *T*: 12.50%
3	Theadom et al., 2018 [26]	Randomized controlled trial	*T*: 24 (62.5) *I*: 12 (58.3) *C*: 12 (66.7)	*T*: 35.9 (11.8) 17 to 56	*I*: 10.42 (7.32) months *C*: 15.09 (10.67) months	*I*: Mild: 11 Moderate: 1 *C*: Mild: 11 Moderate: 1	(1) Sleep disturbance (Pittsburgh Sleep Quality Index, actigraphy sleep onset) (2) Cognitive function: (CNS vital signs online neuropsychological assessment) (3) Quality of life: (Quality of Life after Brain Injury questionnaire)	None	*I*: baseline; 6-week intervention *C*: baseline; 6-week intervention	*I*: 16.67% *C*: 16.67% *T*: 16.67%
4	Nguyen et al., 2017 [34]	Randomized controlled trial	*T*: 24 (33.3) *I*: 13 (30.8) *C*: 11 (36.4)	*I*: 45.53 (13.87) *C*: 41.90 (12.95) *T*: 43.87 (12.95)	77 days to 20.47 years) *I*: 759.15 (714.23) days *C*: 2093.36 (2192.62) days	*I*: Mild: 4 Moderate: 1 Severe: 8 *C*: Mild: 1 Moderate: 1 Severe: 9	(1) Sleep disturbance (Pittsburgh Sleep Quality Index, Insomnia Severity, Index, Epworth Sleepiness Scale) (2) Fatigue: (Brief Fatigue Inventory, Fatigue Severity Scale) (3) Psychological assessment: (Hospital Anxiety and Depression Scale, anxiety and depression)	None	*I*: baseline 2-month intervention; 2-month follow-ups *C*: baseline; 2 months; 2-month follow-ups	*I*: 0% *C*: 0% *T*: 0%
5	Potter et al., 2016 [25]	Randomized controlled trial	*T*: 46 (45.7) *I*: 26 (42.3) *C*: 20 (50.0)	*I*: 40.1 (10.3) *C*: 43.1 (13.1) *T*: 41.4 (11.6)	*I*: 6–12 months: 6 >12, ≤24 months: 6 >24 months: 14 *C*: 6–12 months: 7 >12, ≤24 months: 3 >24 months: 10	*I*: Mild: 12 Moderate: 7 Severe: 6 *C*: Mild: 6 Moderate: 5 Severe: 14	(1) TBI symptom (Rivermead Post-Concussion Symptoms Questionnaire, Brain Injury Community Rehabilitation Outcome Scale, Impact of Event Scale) (2) Psychological assessment: (Hospital Anxiety and Depression Scale, Anxiety and Depression State-Trait Anger Expression Inventory-2) (3) Pain and fatigue (McGill Pain Questionnaire, Checklist of Individual Strength) (4) Quality of life: (Quality of Life Assessment Schedule, European Quality of Life)	None	*I*: baseline 12-week intervention *C*: baseline; 12-week intervention	*I*: 3.85% *C*: 0% *T*: 2.17%
6	Kjeldgaard et al., 2014 [24]	Randomized controlled trial	*T*: 90 (55.6) *I*: 45 *C*: 45	*T*: 34 (11.3)	Not mentioned	*T*: Mild: 90	(1) Pain and headache (basic headache diary, pressure pain thresholds) (2) TBI symptom (Rivermead Post-Concussion Symptoms Questionnaire) (3) Psychological assessment (Symptom Checklist) (4) Quality of life (36-item Short Form Health Survey)	None	*I*: baseline; 16-week intervention *C*: baseline; 16-week intervention	*I*: 22.22% *C*: 17.78% *T*: 20.00%
7	Lah et al., 2019 [32]	Case report	*T*: 5 (20.0)	*T*: 11.8 (0.8)	*T*: 7.4 (2.9) years	Moderate: 2 Severe: 3	(1) Sleep quality (sleep diaries, actigraphy watches, Insomnia Severity Index, Pittsburgh Sleep Quality Index) (2) Fatigue (PedsQL Multidimensional Fatigue Scale)	None	Baseline 4-week intervention 1-week follow-up	After intervention *T*: 28.57% After follow-up *T*: 42.86%
8	Baker et al., 2018 [35]	Case report	*T*: 25 (32.0)	*T*: 29	*T*: 1 month-10 years (average 26 months)	Mild: 25	(1) Pain and headache (migraine frequency, duration, and severity) (2) Quality of life (3) Occupational assessment (current deployment and duty status)	None	Baseline 2-year follow-up	*T*: 0%
9	Lu et al., 2016 [31]	Case report	*T*: 3 (66.7)	*T*: 53.7 (10.1) Case 1: 60 Case 2: 42 Case 3: 59	Case 1: 6 years Case 2: 2 years Case 3: 1 year	Case 1: Mild Case 2: Moderate Case 3: Severe	1. Sleep quality (Insomnia Severity Index, Pittsburgh Sleep Quality Index, Dysfunctional Beliefs and Attitudes about Sleep Scale–Brief Version) 2. Pain (Brief Pain Inventory) (3) Psychological assessment (the Hospital Anxiety and Depression Scale (HADS): anxiety; depression) (4) Fatigue (Multidimensional Assessment of Fatigue-Global Fatigue Index)	None	Baseline 4-week intervention 1-3-month follow-up	*T*: 0%
10	Ouellet and Morin, 2007 [29]	Case report	*T*: 11 (45.5)	*T*: 27.3 (8.5)	*T*: 27.5 (9.7) months	Mild: 1 Mild-moderate: 2 Moderate: 2 Moderate-severe: 3 Severe: 3	(1) Sleep quality (sleep diary, Insomnia Severity Index, Dysfunctional Beliefs and Attitudes about Sleep Scale) (2) Fatigue (Multidimensional Fatigue Inventory) (3) Psychological assessment (Beck Depression/Anxiety Inventory)	None	Baseline 8-10-week intervention 1-3-month follow-up	*T*: 0%
11	Gurr and Coetzer, 2005 [27]	Case report	*T*: 41 (31.7)	*T*: 44.05 Range: 22-78	*T*: 78.7 (108.3) months	Mild: 18 Moderate: 7 Severe: 16	(1) Pain and headache (Headache Disability Inventory, Headache Needs Assessment, Chronic Pain Index) (2) Quality of life (Nottingham Health Profile) (3) Psychological assessment (Hospital Anxiety and Depression Scale)	None	Baseline 14-15-week interventions 12-13-week follow-up	*T*: 51.2%
12	Ouellet and Morin, 2004 [30]	Single-case study	*T*: 1 (0)	*T*: late thirties	1 year	Moderate	(1) Sleep disturbance (sleep diary, polysomnography data, Insomnia Severity Index, Dysfunctional Beliefs and Attitudes about Sleep Scale) (2) Psychological assessment (Beck Anxiety Inventory, Beck Depression Inventory)	None	Baseline 8 weeks of CBT 1-month follow-up 3-month follow-up	*T*: 0

**Table 2:** Cognitive behavioral therapy and control interventions in the included parallel-group trials.

	Author (year)	Cognitive behavioral therapy in the intervention group	Control group intervention	Frequency	Duration
1	McCarty et al., 2021 [33]	Hybrid (telehealth and face-to-face) individualized intervention with care management and enhanced medication consultation	Usual health care	1 hour per week	6 months
2	Tomfohr-Madsen et al., 2019 [28]	Insomnia-specified individualized intervention	Usual health care	45 minutes per week	6 weeks
3	Theadom et al., 2018 [26]	Online individualized intervention with interactive features or suggestions on behavior change	Online education without interactive features or suggestions on behavior change	20 minutes per week	6 weeks
4	Nguyen et al., 2017 [34]	Face-to-face individualized intervention with 30-minute exercise	Usual health care	Moderate exercise 30 minutes 3 to 5 times per week & cognitive behavioral therapy 1 session per week	2 months
5	Potter et al., 2016 [25]	Face-to-face individualized intervention	Waiting list control	1 hour per week	12 weeks
6	Kjeldgaard et al., 2014 [24]	Face-to-face group intervention	Waiting list control	2 hours per week	9 weeks
7	Lah et al., 2019 [32]	Face-to-face insomnia-specified individualized intervention	/	75 minutes per week	4 weeks
8	Baker et al. 2018 [35]	Face-to-face individualized intervention with lifestyle modifications	/	Not mention	2 years
9	Lu et al., 2016 [31]	Insomnia-specified individualized intervention	/	1 hour per week	4 weeks
10	Ouellet and Morin, 2007 [29]	Face-to-face insomnia-specified individualized intervention	/	1 hour per week	8-9 weeks
11	Gurr and Coetzer, 2005 [27]	Face-to-face group relaxation & face-to-face individualized therapy session	/	Group intervention per week for 3 weeks & individualized intervention 30 mins per two weeks for 12 weeks	14-15 weeks
12	Ouellet and Morin, 2004 [30]	Face-to-face insomnia-specified individualized intervention	/	1 session per week	8 weeks

**Table 3:** The Cochrane Collaboration's tool for assessing risk of bias for methodological assessment.

Article (year)	Random sequence generation	Allocation concealment	Blinding of participants and personnel	Blinding of outcome assessments	Incomplete outcome data	Selective reporting	Other bias
McCarty et al., 2021	Low	Unclear	High	Low	Low	High	Unclear
Tomfohr-Madsen et al., 2019	Low	Unclear	High	Low	High	Low	Unclear
Theadom et al., 2017 [26]	Low	Low	Unclear	Unclear	High	Low	Unclear
Nguyen et al., 2017 [34]	Low	Unclear	High	Low	Low	Low	Unclear
Potter et al., 2016 [25]	Unclear	Unclear	High	High	Low	High	Unclear
Kjeldgaard et al., 2014 [24]	Low	Low	High	High	High	High	Unclear

## Supplementary Materials

Supplementary Materials Appendix S1: the search strategies for the databases. Appendix S2: results of GRADE criteria.
