# Supplementary material for: Effects of Cognitive Behavioral Therapy on Pain and Sleep in Adults with Traumatic Brain Injury: A Systematic Review and Meta-Analysis
Source: Neural Plast. 2021 Nov 11;2021:6552246. doi: 10.1155/2021/6552246 (PMC8601855; doi:10.1155/2021/6552246)
Supplement: Supplementary Materials — Appendix S1: the search strategies for the databases. Appendix S2: results of GRADE criteria. [file 6552246.f1.zip › Appendix S1 the search strategies for the databases.docx]

**Search strategies for all databases July 26^th^, 2021**

1. Search Strategy for PubMed: 59 items

#1 “parallel”[Text Word] OR “controlled trial*”[Text Word] OR “random*”[Text Word] OR “randomi*”[Text Word] OR “intervention*”[Text Word]

#2 “clinical trial”[PT]

#3 #1 OR #2

#4  animals NOT humans

#5 #3 NOT #4

#6 “Brain Injuries, Traumatic”[Mesh] OR “Traumatic Brain Injur*”[All Fields] OR “TBI”[All Fields] OR “TBIs”[All Fields] OR “Brain Injury, Traumatic”[All Fields] OR “Brain Injuries, Traumatic”[All Fields] OR “brain injur*”[ All Fields] OR “brain trauma*”[ All Fields] OR “brain damage”[ All Fields] OR “brain lesion*”[ All Fields]OR “Traumatic Encephalopath*”[ All Fields] OR “Encephalopathy, Traumatic”[ All Fields] OR “head injur*”[All Fields] OR “head trauma*”[All Fields] OR “head damage”[All Fields] OR “head lesion*”[All Fields]OR “craniocerebral trauma*”[ All Fields] OR “craniocerebral injur*”[ All Fields] OR “craniocerebral damage”[ All Fields] OR “craniocerebral lesion*”[ All Fields] OR “cerebral injur*”[ All Fields] OR “cerebral trauma*”[ All Fields] OR “cerebral damage”[ All Fields] OR “cerebral lesion*”[ All Fields] OR “concussion*”[ All Fields]

#7 “Cognitive Behavioral Therapy”[Mesh] OR”CBT”[All Fields] OR”CBTs”[All Fields] OR “Behavioral Therapies, Cognitive”[All Fields] OR “Behavioral Therapy, Cognitive”[All Fields] OR “Therapies, Cognitive Behavioral”[All Fields] OR “Therapy, Cognitive Behavioral”[All Fields] OR “Therapy, Cognitive Behavior”[All Fields] OR “Cognitive Behavior Therapy”[All Fields] OR “Cognitive-Behavior Therapy”[All Fields] OR “Cognitive Therapy”[All Fields] OR “Behavior Therapy, Cognitive”[All Fields] OR “Behavior Therapies, Cognitive”[All Fields] OR “Cognitive Behavior Therapies”[All Fields] OR “Therapies, Cognitive Behavior”[All Fields] OR “Cognitive Psychotherapy”[All Fields] OR “Cognitive Psychotherapies”[All Fields] OR “Psychotherapies, Cognitive”[All Fields] OR “Psychotherapy, Cognitive”[All Fields] OR “Therapy, Cognitive”[All Fields] OR “Cognitive Therapies”[All Fields] OR “Therapies, Cognitive”[All Fields] OR “Cognition Therapy”[All Fields] OR “Therapy, Cognition”[All Fields] OR “Cognition Therapies”[All Fields] OR “cognitive behaviour therap*”[All Fields] OR “cognitive behavioural therap*”[All Fields] OR “cognitive-behavioural therap*”[All Fields] OR “Behavioural Therapies, Cognitive”[All Fields] OR “Behavioural Therapy, Cognitive”[All Fields] OR “Therapies, Cognitive Behavioural”[All Fields] OR “Therapy, Cognitive Behavioural”[All Fields] OR “Therapy, Cognitive Behaviour”[All Fields] OR “Cognitive Behaviour Therapy”[All Fields] OR “Cognitive Therapy”[All Fields] OR “Behaviour Therapy, Cognitive”[All Fields] OR “Cognitive Behaviour Therapies”[All Fields] OR “Therapies, Cognitive Behaviour”[All Fields]

#8 “Sleep”[Mesh] OR “Sleep*”[All Fields] OR “Sleep Wake Disorders”[Mesh] OR “Insomnia”[All Fields] OR “Dysomnias”[All Fields] OR “Parasomnias”[All Fields] OR “Hypersomnia”[All Fields] OR “Somnolence”[All Fields] OR “Circadian Rhythm”[All Fields]

#9 "Pain"[Mesh] OR “Pain*”[All Fields] OR "Ache*"[All Fields] OR "Physical Suffering*"[All Fields] OR "Suffering, Physical"[All Fields] OR "Cervicalgia*"[All Fields] OR "Cervicodynia*"[All Fields] OR "Neckache*"[All Fields] OR "Headache"[Mesh] OR “Headache*”[All Fields] OR “Cephalodynia*”[All Fields] OR “Cephalalgia*”[All Fields] OR “Cephalgia*”[All Fields]

#10 #8 OR #9

#11 #5 AND #6 AND #7 AND #10

2. Search Strategy for EMBASE: 111 items

#1 ‘Parallel’/exp OR ‘Observational’/exp OR ‘Cross-Sectional’/exp OR ‘Pre–Post’/exp OR ‘Before-After’/exp OR ‘Controlled Trial*’/exp OR ‘Random*’/exp OR ‘ Randomi*’/exp OR ‘Intervention*’/exp

#2 Random*:ab,ti OR Parallel:ab,ti OR Pre-Post:ab,ti OR Before-After:ab,ti

#3 #1 OR #2

#4 ‘Traumatic Brain Injur*’ OR ‘Brain Injury, Traumatic’ OR ‘Brain Injuries, Traumatic’ OR ‘Traumatic Brain Injury’/exp OR TBI OR TBIs OR ‘Head Injur*’ OR ‘Injury, Head’ OR ‘Injuries, Head’ OR ‘Head Injury’/exp OR ‘Brain Injur*’ OR ‘Injury, Brain’ OR ‘Injuries, Brain’ OR ‘Brain Injury’/exp OR ‘Brain Lesion*’ OR ‘Lesion, Brain’ OR ‘Lesions, Brain’ OR ‘Brain Damage’ OR ‘Damage, Brain’ OR ‘Brain Trauma*’ OR ‘Trauma, Brain’ OR ‘Traumas, Brain’ OR ‘Brain Laceration*’ OR ‘Laceration, Brain’ OR ‘Lacerations, Brain’ OR ‘Cerebral Injur*’ OR ‘ Injury, Cerebral’ OR ‘ Injuries, Cerebral’ OR ‘Craniocerebral Trauma*’ OR ‘Trauma*, Craniocerebral’ OR ‘Traumas, Craniocerebral’ OR ‘Craniocerebral Injur*’ OR ‘Injury, Craniocerebral’ OR ‘Injuries, Craniocerebral’

#5 ‘Cognitive Behavioral Therapy’/exp OR CBT OR CBTs OR ‘Cognitive Behavioral Therap*’ OR ‘Cognitive-Behavioral Therap*’ OR ‘Cognitive Behavior Therap*’ OR ‘Cognition Behavioral Therap*’ OR ‘Cognition Behavior Therap*’ OR ‘Cognitive Behavioral Treatment’ OR ‘Cognitive Behavior Treatment’ OR ‘Cognition Behavioral Treatment’ OR ‘Cognition Behavior Treatment’ OR ‘Cognitive Behavioural Therap*’ OR ‘Cognitive-Behavioural Therap*’ OR ‘Cognitive Behaviour Therap*’ OR ‘Cognition Behavioural Therap*’ OR ‘Cognition Behaviour Therap*’ OR ‘Cognitive Behavioural Treatment’ OR ‘Cognitive Behaviour Treatment’ OR ‘Cognition Behavioural Treatment’ OR ‘Cognition Behaviour Treatment’

#6  ‘Sleep’/exp OR ‘Sleep*’ OR ‘Sleep Disorder’/exp OR ‘Insomnia’ OR ‘Dysomnias’ OR ‘Parasomnias’ OR ‘Hypersomnia’ OR ‘Somnolence’ OR ‘Circadian Rhythm’

#7 ‘Pain’/exp OR ‘Pain*’ OR ‘Ache*’ OR ‘Physical Suffering*’ OR ‘Suffering, Physical’ OR ‘Neck Pain’/exp OR ‘Cervicalgia*’ OR ‘Cervicodynia*’ OR ‘Neckache*’ OR ‘Headache’/exp OR ‘Headache*’ OR ‘Cephalodynia*’ OR ‘Cephalalgia*’ OR ‘Cephalgia*’

#8 #6 OR #7

#9 #3 AND #4 AND #5 AND #8

3. Search Strategy for Cochrane Library 398 items

#1 Parallel OR Pre-Post OR Before-After OR Controlled Trial* OR Random* OR Randomi* OR Intervention*

#2 Randomized Controlled Trial:pt OR Clinical Trial:pt

#3 #1 OR #2

#4 Traumatic Brain Injur*:ti,ab,kw OR TBI:ti,ab,kw OR TBIs:ti,ab,kw OR Brain Injury, Traumatic:ti,ab,kw OR brain injur*:ti,ab,kw OR brain trauma*:ti,ab,kw OR brain damage:ti,ab,kw OR brain lesion*:ti,ab,kw OR Traumatic Encephalopath*:ti,ab,kw OR Encephalopathy, Traumatic:ti,ab,kw OR head injur*:ti,ab,kw OR head trauma*:ti,ab,kw OR head damage:ti,ab,kw OR head lesion*OR craniocerebral trauma*:ti,ab,kw OR craniocerebral injur*:ti,ab,kw OR craniocerebral damage:ti,ab,kw OR craniocerebral lesion*:ti,ab,kw OR cerebral injur*:ti,ab,kw OR cerebral trauma*:ti,ab,kw OR cerebral damage:ti,ab,kw OR cerebral lesion*:ti,ab,kw OR concussion*:ti,ab,kw

#5 Cognitive Behavioral Therapy:ti,ab,kw OR CBT:ti,ab,kw OR CBTs:ti,ab,kw OR Behavioral Therapies, Cognitive:ti,ab,kw OR Behavioral Therapy, Cognitive:ti,ab,kw OR Therapies, Cognitive Behavioral:ti,ab,kw OR Therapy, Cognitive Behavioral:ti,ab,kw OR Therapy, Cognitive Behavior:ti,ab,kw OR Cognitive Behavior Therapy:ti,ab,kw OR Cognitive Behavior Therapy:ti,ab,kw OR Cognitive Therapy:ti,ab,kw OR Behavior Therapy, Cognitive:ti,ab,kw OR Behavior Therapies, Cognitive:ti,ab,kw OR Cognitive Behavior Therapies:ti,ab,kw OR Therapies, Cognitive Behavior:ti,ab,kw OR Cognitive Psychotherapy:ti,ab,kw OR Cognitive Psychotherapies:ti,ab,kw OR Psychotherapies, Cognitive:ti,ab,kw OR Psychotherapy, Cognitive:ti,ab,kw OR Therapy, Cognitive:ti,ab,kw OR Cognitive Therapies:ti,ab,kw OR Therapies, Cognitive:ti,ab,kw OR Cognition Therapy:ti,ab,kw OR Therapy, Cognition:ti,ab,kw OR Cognition Therapies:ti,ab,kw OR Cognitive Behaviour Therap*:ti,ab,kw OR Cognitive Behavioural Therap*:ti,ab,kw OR Behavioural Therapies, Cognitive:ti,ab,kw OR Behavioural Therapy, Cognitive:ti,ab,kw OR Therapies, Cognitive Behavioural:ti,ab,kw OR Therapy, Cognitive Behavioural:ti,ab,kw OR Therapy, Cognitive Behaviour:ti,ab,kw OR Cognitive Behaviour Therapy:ti,ab,kw OR Cognitive Therapy:ti,ab,kw OR Behaviour Therapy, Cognitive:ti,ab,kw OR Cognitive Behaviour Therapies:ti,ab,kw OR Therapies, Cognitive Behaviour:ti,ab,kw

#6 Sleep*:ti,ab,kw OR Insomnia:ti,ab,kw OR Dysomnias:ti,ab,kw OR Parasomnias:ti,ab,kw OR Hypersomnia:ti,ab,kw OR Somnolence:ti,ab,kw OR Circadian Rhythm:ti,ab,kw

#7 Pain*:ti,ab,kw OR Ache*:ti,ab,kw OR Physical Suffering*:ti,ab,kw OR Cervicalgia*:ti,ab,kw OR Cervicodynia*:ti,ab,kw OR Neckache*:ti,ab,kw OR Headache*:ti,ab,kw OR Cephalodynia*:ti,ab,kw OR Cephalalgia*:ti,ab,kw OR Cephalgia*:ti,ab,kw

#8 #6 OR #7

#9 #3 AND #4 AND #5 AND #8

4. Search Strategy for Web of Science 95 items

#1 TS=(parallel OR observational OR cross-sectional OR pre–post OR before-after OR controlled trial* OR random* OR randomi* OR intervention*)

#2 TS=(“traumatic brain injur*” OR “TBI” OR “TBIs” OR “brain injur*” OR “brain trauma*” OR “brain damage” OR “brain lesion*” OR “concussion*” OR “ head injur*” OR “ head trauma*” OR “ head damage” OR “ head lesion*” OR “craniocerebral injur*” OR “craniocerebral trauma*” OR “craniocerebral lesion*” OR “craniocerebral damage” OR “cerebral injur*” OR “cerebral trauma*” OR “cerebral lesion*” OR “cerebral damage” OR “cerebral contusion and laceration*”)

#3 TS=(“Cognitive Behavioral Therap*” OR “Cognitive-Behavioral Therap*” OR “Cognitive Behavior Therap*” OR “CBT” OR “CBTs” OR “Cognitive Behavioural Therap*” OR “Cognitive-Behavioural Therap*” OR “Cognitive Behaviour Therap*” OR “Cognitive Therap*” OR “Cognitive Psychotherap*” OR “Cognition Therap*”)

#4 TS=( “Sleep*” OR “Insomnia” OR “Dysomnias” OR “Parasomnias” OR “Hypersomnia” OR “Somnolence” OR “Circadian Rhythm”)

#5 TS=(“Pain*” OR “Ache*” OR “Physical Suffering*” OR “Neckache*” OR “Cervicalgia*” OR “Cervicodynia*” OR “Headache*” OR “Cephalodynia*” OR “Cephalalgia*” OR “Cephalgia*”)

#6 #4 OR #5

#7 #1 AND #2 AND #3 AND #6

5. Search Strategy for CINAHL (Ebsco) 74 items

S1 MH(“random assignment” OR “placebos” OR “placebo effect” OR “single-blind studies” OR “double-blind studies” OR “triple-blind studies” OR “randomized controlled trials” OR “comparative studies” OR “evaluation research” OR “prospective studies” OR “crossover design” OR “prospective studies” OR “clinical trials” OR “clinical trial registry”)

S2 TX (random* OR allocation OR placebo* OR “single blind” OR “double blind” OR “comparative study” OR “evaluation stud*” OR “follow-up stud*” OR “prospective stud*” OR “cross-over stud*” OR control* OR prospective* OR volunteer* OR “RCT” OR “clinical trial*”)

S3 PT (“randomized controlled trial” OR “clinical trial”)

S4 S1 OR S2 OR S3

S5 TX(“traumatic brain injur*” OR “TBI” OR “TBIs” OR “brain injur*” OR “brain trauma*” OR “brain damage” OR “brain lesion*” OR “concussion*” OR “ head injur*” OR “ head trauma*” OR “ head damage” OR “ head lesion*” OR “craniocerebral injur*” OR “craniocerebral trauma*” OR “craniocerebral lesion*” OR “craniocerebral damage” OR “cerebral injur*” OR “cerebral trauma*” OR “cerebral lesion*” OR “cerebral damage” OR “cerebral contusion and laceration*”)

S6 TX(“Cognitive Behavioral Therap*” OR “Cognitive Behavior Therap*” OR “CBT” OR “CBTs” OR “Cognitive Behavioural Therap*” OR “Cognitive Behaviour Therap*” OR “Cognitive Therap*” OR “Cognitive Psychotherap*” OR “Cognition Therap*”)

S7 TX(“Pain*” OR “Ache*” OR “Acute Pain*” OR “Physical Suffering*” OR “Suffering, Physical” OR “Cervicalgia*” OR “Cervicodynia*” OR “Neckache*” OR “Cephalodynia*” OR “Cephalalgia*” OR “Cephalgia*” )

S8 TX(“Sleep*” OR “Insomnia” OR “Dysomnias” OR “Parasomnias” OR “Hypersomnia” OR “Somnolence” OR “Circadian Rhythm”)

S9 S7 OR S8

S10 S4 AND S5 AND S6 AND S9
