# Supplementary material for: Effects of Cognitive Behavioral Therapy on Pain and Sleep in Adults with Traumatic Brain Injury: A Systematic Review and Meta-Analysis
Source: Neural Plast. 2021 Nov 11;2021:6552246. doi: 10.1155/2021/6552246 (PMC8601855; doi:10.1155/2021/6552246)
Supplement: Supplementary Materials — Appendix S1: the search strategies for the databases. Appendix S2: results of GRADE criteria. [file 6552246.f1.zip › Appendix S2 results of GRADE criteria..doc]

| **Effects of cognitive behavioral therapy on pain and sleep in adults with traumatic brain injuries: a systematic review and meta-analysis of randomized controlled trials** | | | | | |
| --- | --- | --- | --- | --- | --- |
| **Patient or population:** Patients with traumatic brain injuries **Settings:** Clinical **Intervention:** Cognitive behavioral therapy  **Comparison:** No treatment or non-cognitive behavioral therapy treatment | | | | | |
| **Outcomes** | **Illustrative comparative risks* (95% CI)** | | **Relative effect (95% CI)** | **No of Participants (studies)** | **Quality of the evidence (GRADE)** |
|  | Assumed risk | Corresponding risk |  |  |  |
|  | **Control** | **Cognitive behavior therapy** |  |  |  |
| **Self-reported sleep quality** the Pittsburgh self-reported sleep quality index (PSQI) Follow-up: median 6 weeks |  | The mean self-reported sleep quality in the intervention groups was **2.30 lower** (3.45 to 1.15 lower) |  | 72 (3 studies) | ⊕⊕⊝⊝ **low**^1,2,4,5,6^ |
| **Self-reported sleep quality** the Insomnia Severity Index (ISI) Follow-up: median 7 weeks |  | The mean self-reported sleep quality in the intervention groups was **5.12 lower** (9.69 to 0.55 lower) |  | 48 (2 studies) | ⊕⊝⊝⊝ **very low**^1,3,4,5,6^ |
| *The basis for the **assumed risk** (e.g. the median control group risk across studies) is provided in footnotes. The **corresponding risk** (and its 95% confidence interval) is based on the assumed risk in the comparison group and the **relative effect** of the intervention (and its 95% CI). **CI:** Confidence interval; | | | | | |
| GRADE Working Group grades of evidence **High quality:** Further research is very unlikely to change our confidence in the estimate of effect.  **Moderate quality:** Further research is likely to have an important impact on our confidence in the estimate of effect and may change the estimate. **Low quality:** Further research is very likely to have an important impact on our confidence in the estimate of effect and is likely to change the estimate. **Very low quality:** We are very uncertain about the estimate. | | | | | |
| ^1^ >25% of the participants was from studies with high risk of bias. ^2^. *I^2^* =0%. ^3^ Participants in the pooled studies consisted of both adolescents and adults. ^4^ There were less than 100 participants in total. ^5^ No funnel plots could be used to assess publication bias. ^6^ *I^2^* > 50% | | | | | |
